# 2-Chloro­quinoline-3-carbaldehyde

**DOI:** 10.1107/S1600536809040665

**Published:** 2009-10-10

**Authors:** F. Nawaz Khan, R. Subashini, Rajesh Kumar, Venkatesha R. Hathwar, Seik Weng Ng

**Affiliations:** a Chemistry Division, School of Science and Humanities, VIT University, Vellore 632 014, Tamil Nadu, India; b Solid State and Structural Chemistry Unit, Indian Institute of Science, Bangalore 560 012, Karnataka, India; c Department of Chemistry, University of Malaya, 50603 Kuala Lumpur, Malaysia

## Abstract

The quinolinyl fused ring system of the title compound, C_10_H_6_ClNO, is planar (r.m.s. deviation = 0.018 Å); the formyl group is slightly bent out of the plane of the fused ring system [C—C—C—O torsion angle = 8.2 (3)°].

## Related literature

For the synthesis of 2-chloro­quinoline-3-carbaldehyde by Vilsmeier–Haack cyclization, see: Ali *et al.* (2001 ▶, 2002 ▶); Mogilaiah *et al.* (2002 ▶); Pawar *et al.* (1990 ▶); Srivastava & Singh (2005 ▶). For a review of the synthesis of quinolines by this reaction, see: Meth-Cohn (1993 ▶).
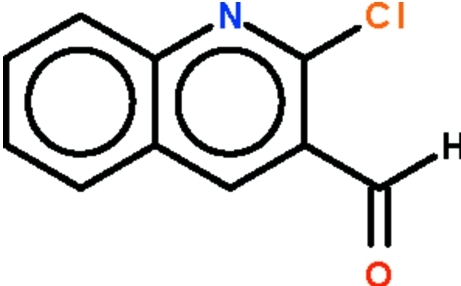


## Experimental

### 

#### Crystal data

C_10_H_6_ClNO
*M* *_r_* = 191.61
Monoclinic, 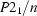

*a* = 11.8784 (9) Å
*b* = 3.9235 (3) Å
*c* = 18.1375 (12) Å
β = 101.365 (4)°
*V* = 828.72 (10) Å^3^
*Z* = 4
Mo *K*α radiation
μ = 0.41 mm^−1^
*T* = 290 K
0.24 × 0.18 × 0.14 mm

#### Data collection

Bruker SMART area-detector diffractometer
Absorption correction: multi-scan (*SADABS*; Sheldrick, 1996 ▶) *T*_min_ = 0.908, *T*_max_ = 0.945
6886 measured reflections
1889 independent reflections
1626 reflections with *I* > 2σ(*I*)
*R*_int_ = 0.021

#### Refinement

*R*[*F* ^2^ > 2σ(*F* ^2^)] = 0.033
*wR*(*F* ^2^) = 0.145
*S* = 1.19
1889 reflections
118 parameters
H-atom parameters constrained
Δρ_max_ = 0.36 e Å^−3^
Δρ_min_ = −0.29 e Å^−3^

### 

Data collection: *SMART* (Bruker, 2004 ▶); cell refinement: *SAINT* (Bruker, 2004 ▶); data reduction: *SAINT*; program(s) used to solve structure: *SHELXS97* (Sheldrick, 2008 ▶); program(s) used to refine structure: *SHELXL97* (Sheldrick, 2008 ▶); molecular graphics: *X-SEED* (Barbour, 2001 ▶); software used to prepare material for publication: *publCIF* (Westrip, 2009 ▶).

## Supplementary Material

Crystal structure: contains datablocks global, I. DOI: 10.1107/S1600536809040665/tk2549sup1.cif

Structure factors: contains datablocks I. DOI: 10.1107/S1600536809040665/tk2549Isup2.hkl

Additional supplementary materials:  crystallographic information; 3D view; checkCIF report

## Figures and Tables

**Fig. 1.**
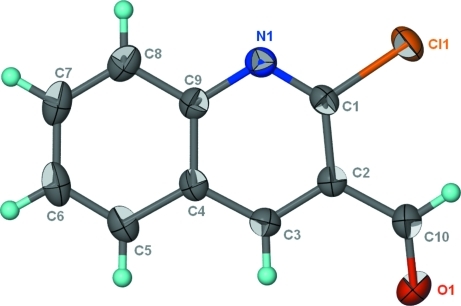
Thermal ellipsoid plot (Barbour, 2001) of C10H6ClNO at the 50% probability level; hydrogen atoms are drawn as spheres of arbitrary radius.

## supplementary crystallographic information

### Experimental

A Vilsmeier-Haack adduct prepared from phosphorus oxytrichloride (6.5 ml, 70 mmol) and *N*,*N*-dimethylformamide (2.3 ml, 30 mmol) at 273 K was added to *N*-phenylacetamide (1.35 g, 10 mmol), heated at 353 K for 15 h. The mixture was then poured onto ice, and the white product was collected and dried. The compound was purified by recrystallization from a petroleum ether/ethyl acetate mixture.

### Refinement

Carbon-bound H-atoms were placed in calculated positions (C–H 0.93 Å) and were included in the refinement in the riding model approximation with *U*_iso_(H) set to 1.2*U*_eq_(C).

### Crystal data

**Table:**

C_10_H_6_ClNO	*F*(000) = 392
*M**_r_* = 191.61	*D*_x_ = 1.536 Mg m^−^^3^
Monoclinic, *P*2_1_/*n*	Mo *K*α radiation, λ = 0.71073 Å
Hall symbol: -P 2yn	Cell parameters from 781 reflections
*a* = 11.8784 (9) Å	θ = 2.1–24.3°
*b* = 3.9235 (3) Å	µ = 0.41 mm^−^^1^
*c* = 18.1375 (12) Å	*T* = 290 K
β = 101.365 (4)°	Block, colorless
*V* = 828.72 (10) Å^3^	0.24 × 0.18 × 0.14 mm
*Z* = 4	

### Data collection

**Table:**

Bruker SMART area-detector diffractometer	1889 independent reflections
Radiation source: fine-focus sealed tube	1626 reflections with *I* > 2σ(*I*)
graphite	*R*_int_ = 0.021
φ and ω scans	θ_max_ = 27.5°, θ_min_ = 1.9°
Absorption correction: multi-scan (*SADABS*; Sheldrick, 1996)	*h* = −15→15
*T*_min_ = 0.908, *T*_max_ = 0.945	*k* = −3→5
6886 measured reflections	*l* = −23→23

### Refinement

**Table:**

Refinement on *F*^2^	Primary atom site location: structure-invariant direct methods
Least-squares matrix: full	Secondary atom site location: difference Fourier map
*R*[*F*^2^ > 2σ(*F*^2^)] = 0.033	Hydrogen site location: inferred from neighbouring sites
*wR*(*F*^2^) = 0.145	H-atom parameters constrained
*S* = 1.19	*w* = 1/[σ^2^(*F*_o_^2^) + (0.0923*P*)^2^ + 0.077*P*] where *P* = (*F*_o_^2^ + 2*F*_c_^2^)/3
1889 reflections	(Δ/σ)_max_ = 0.001
118 parameters	Δρ_max_ = 0.36 e Å^−^^3^
0 restraints	Δρ_min_ = −0.29 e Å^−^^3^

### Fractional atomic coordinates and isotropic or equivalent isotropic displacement parameters (Å^2^)

**Table:**

	*x*	*y*	*z*	*U*_iso_*/*U*_eq_	
Cl1	0.24556 (4)	0.22180 (12)	0.67041 (2)	0.0464 (2)	
O1	0.51095 (11)	0.8482 (4)	0.61287 (8)	0.0550 (4)	
N1	0.15508 (12)	0.2731 (3)	0.52950 (9)	0.0353 (3)	
C1	0.24476 (14)	0.3582 (4)	0.57833 (9)	0.0319 (4)	
C2	0.34039 (13)	0.5468 (4)	0.56384 (9)	0.0323 (4)	
C3	0.33695 (13)	0.6369 (4)	0.49061 (9)	0.0334 (4)	
H3	0.3977	0.7585	0.4781	0.040*	
C4	0.24263 (13)	0.5477 (4)	0.43407 (9)	0.0324 (4)	
C5	0.23434 (16)	0.6341 (5)	0.35765 (10)	0.0419 (4)	
H5	0.2944	0.7483	0.3424	0.050*	
C6	0.13859 (17)	0.5504 (5)	0.30635 (10)	0.0481 (5)	
H6	0.1334	0.6077	0.2560	0.058*	
C7	0.04727 (17)	0.3774 (6)	0.32911 (11)	0.0493 (5)	
H7	−0.0180	0.3243	0.2935	0.059*	
C8	0.05247 (16)	0.2861 (5)	0.40222 (12)	0.0436 (4)	
H8	−0.0082	0.1699	0.4162	0.052*	
C9	0.15087 (13)	0.3697 (4)	0.45629 (9)	0.0325 (4)	
C10	0.43810 (15)	0.6537 (5)	0.62315 (10)	0.0407 (4)	
H10	0.4432	0.5645	0.6712	0.049*	

### Atomic displacement parameters (Å^2^)

**Table:**

	*U*^11^	*U*^22^	*U*^33^	*U*^12^	*U*^13^	*U*^23^
Cl1	0.0587 (3)	0.0522 (3)	0.0308 (3)	−0.00331 (19)	0.0147 (2)	0.00444 (16)
O1	0.0435 (8)	0.0681 (10)	0.0499 (8)	−0.0161 (6)	0.0004 (6)	0.0003 (7)
N1	0.0363 (7)	0.0355 (7)	0.0355 (8)	−0.0016 (5)	0.0107 (6)	−0.0029 (5)
C1	0.0381 (8)	0.0310 (8)	0.0283 (7)	0.0020 (6)	0.0107 (6)	0.0004 (6)
C2	0.0335 (8)	0.0312 (8)	0.0321 (8)	0.0024 (6)	0.0062 (6)	−0.0007 (6)
C3	0.0329 (8)	0.0332 (8)	0.0349 (8)	−0.0001 (6)	0.0089 (6)	0.0018 (7)
C4	0.0362 (8)	0.0317 (8)	0.0299 (8)	0.0056 (6)	0.0077 (6)	−0.0005 (6)
C5	0.0489 (10)	0.0444 (9)	0.0332 (9)	0.0076 (8)	0.0101 (7)	0.0039 (7)
C6	0.0591 (11)	0.0544 (11)	0.0289 (8)	0.0166 (9)	0.0039 (8)	−0.0021 (8)
C7	0.0454 (10)	0.0560 (11)	0.0407 (10)	0.0110 (8)	−0.0058 (8)	−0.0141 (9)
C8	0.0356 (9)	0.0467 (10)	0.0466 (11)	0.0012 (7)	0.0037 (8)	−0.0120 (8)
C9	0.0331 (8)	0.0320 (8)	0.0329 (8)	0.0032 (6)	0.0072 (6)	−0.0052 (6)
C10	0.0414 (9)	0.0459 (10)	0.0332 (9)	−0.0003 (7)	0.0031 (7)	0.0017 (7)

### Geometric parameters (Å, °)

**Table:**

Cl1—C1	1.7519 (16)	C4—C9	1.418 (2)
O1—C10	1.196 (2)	C5—C6	1.360 (3)
N1—C1	1.288 (2)	C5—H5	0.9300
N1—C9	1.372 (2)	C6—C7	1.409 (3)
C1—C2	1.423 (2)	C6—H6	0.9300
C2—C3	1.367 (2)	C7—C8	1.363 (3)
C2—C10	1.479 (2)	C7—H7	0.9300
C3—C4	1.406 (2)	C8—C9	1.409 (2)
C3—H3	0.9300	C8—H8	0.9300
C4—C5	1.411 (2)	C10—H10	0.9300
			
C1—N1—C9	117.48 (14)	C5—C6—C7	120.28 (17)
N1—C1—C2	126.15 (15)	C5—C6—H6	119.9
N1—C1—Cl1	115.14 (12)	C7—C6—H6	119.9
C2—C1—Cl1	118.71 (12)	C8—C7—C6	121.46 (17)
C3—C2—C1	116.22 (14)	C8—C7—H7	119.3
C3—C2—C10	120.14 (15)	C6—C7—H7	119.3
C1—C2—C10	123.62 (15)	C7—C8—C9	119.23 (18)
C2—C3—C4	120.74 (14)	C7—C8—H8	120.4
C2—C3—H3	119.6	C9—C8—H8	120.4
C4—C3—H3	119.6	N1—C9—C8	118.45 (15)
C3—C4—C5	123.22 (15)	N1—C9—C4	121.83 (14)
C3—C4—C9	117.52 (14)	C8—C9—C4	119.71 (16)
C5—C4—C9	119.24 (15)	O1—C10—C2	123.76 (16)
C6—C5—C4	120.07 (17)	O1—C10—H10	118.1
C6—C5—H5	120.0	C2—C10—H10	118.1
C4—C5—H5	120.0		
			
C9—N1—C1—C2	0.6 (2)	C5—C6—C7—C8	−0.8 (3)
C9—N1—C1—Cl1	−179.13 (11)	C6—C7—C8—C9	0.7 (3)
N1—C1—C2—C3	−1.8 (2)	C1—N1—C9—C8	−178.61 (14)
Cl1—C1—C2—C3	177.90 (11)	C1—N1—C9—C4	1.8 (2)
N1—C1—C2—C10	176.39 (16)	C7—C8—C9—N1	−179.43 (15)
Cl1—C1—C2—C10	−3.9 (2)	C7—C8—C9—C4	0.1 (3)
C1—C2—C3—C4	0.6 (2)	C3—C4—C9—N1	−2.9 (2)
C10—C2—C3—C4	−177.67 (14)	C5—C4—C9—N1	178.69 (15)
C2—C3—C4—C5	179.92 (15)	C3—C4—C9—C8	177.57 (15)
C2—C3—C4—C9	1.5 (2)	C5—C4—C9—C8	−0.9 (2)
C3—C4—C5—C6	−177.55 (16)	C3—C2—C10—O1	8.2 (3)
C9—C4—C5—C6	0.8 (3)	C1—C2—C10—O1	−170.0 (2)
C4—C5—C6—C7	0.0 (3)		
